# Risk and mortality of testicular cancer in patients with neurodevelopmental or other psychiatric disorders

**DOI:** 10.1038/s41416-023-02260-8

**Published:** 2023-04-24

**Authors:** Anna K. Jansson, Jonas Söderling, Johan Reutfors, Anna Thor, Camilla Sköld, Gabriella Cohn-Cedermark, Olof Ståhl, Karin E. Smedby, Andreas Pettersson, Ingrid Glimelius

**Affiliations:** 1 Department of Immunology, Genetics & Pathology, Uppsala University, Uppsala, Sweden; 2 Clinical Epidemiology Division, Department of Medicine Solna, Karolinska Institutet, Stockholm, Sweden; 3 Division of Urology, Department of Clinical Science, Intervention and Technology, Karolinska Institutet, Stockholm, Sweden; 4 Department of Oncology-Pathology, Karolinska Institutet, Stockholm, Sweden; 5 Genitourinary Oncology Unit, Department of Pelvic Cancer, Karolinska University Hospital, Stockholm, Sweden; 6 Department of Oncology, Skåne University Hospital, Lund, Sweden

**Keywords:** Germ cell tumours, Cancer epidemiology, Germ cell tumours

## Abstract

**Background:**

Both testicular germ cell tumours (TGCT) and neurodevelopmental disorders are associated with urogenital malformations. Few studies have investigated the association between psychiatric disorders and TGCT. We investigated whether history of any psychiatric or neurodevelopmental disorder is associated with increased risk or mortality of TGCT.

**Method:**

This is a nested case–control study including 6166 TGCT patients diagnosed during 1992–2014, individually matched for age and calendar period to 61,660 controls. We calculated odds ratios (ORs) for the association between type of psychiatric diagnoses and TGCT risk. Among the cases, we used a cohort design and calculated hazard ratios (HRs) of the association between psychiatric diagnose and all-cause and TGCT-specific death.

**Results:**

History of a neurodevelopmental disorder (attention deficit hyperactivity disorder, autism spectrum disorder and intellectual disabilities) was associated with an increased risk of seminoma (OR: 1.54; 1.09–2.19). Seminoma patients with neurodevelopmental disorders were younger (34 versus 38 years, *p* = 0.004) and had more stage IV disease (5.4% versus 1.2%) than those without. Psychiatric history overall was not associated with TGCT. Patient history of any psychiatric disorder was associated with an increased all-cause and TGCT-specific death.

**Conclusions:**

We report an association between neurodevelopmental disorders and testicular seminoma, and an increased TGCT-specific mortality for TGCT patients with psychiatric disorders.

## Introduction

Testicular cancer is the most common cancer in young men, with an incidence in Sweden of 7.4/100,000 [1]. The incidence increases in Europe and several other parts of the world [2, 3]. Ninety-five percent of all testicular cancers are testicular germ cell tumours (TGCT), of which 55–60% are seminomas and 40–45% non-seminomas [4].

The cause of testicular cancer is not known, but some risk factors have been identified, including cryptorchidism, hypospadias, inguinal hernias, previous testicular cancer and family history of testicular cancer [4]. TGCT have a high heritability and genome-wide association studies have identified common variation associated with TGCT susceptibility, which is estimated to account for up to 44% of the disease heritability [5]. Furthermore, low birth weight and short gestational age seem to be associated with an increased risk of TGCT [6]. Some risk factors during adolescence and adulthood have also been described, such as having an occupation as a fire-fighter, working with aircraft maintenance [7] and marijuana smoking [8].

There are indications that the first steps of TGCT oncogenesis occur in utero and it has been hypothesised that cryptorchidism, hypospadias, poor semen quality and TGCT form part of a testicular dysgenesis syndrome with a common origin in fetal life [9, 10]. Both cryptorchidism and hypospadias have been associated with neurodevelopmental disorders such as autism spectrum disorder (ASD), attention deficit hyperactivity disorder (ADHD) and intellectual disabilities (ID) and other behavioural/emotional disorders with onset in childhood [11, 12]. Furthermore, ASD, ADHD and ID share some of the risk factors of TGCT, for example, low birth weight [13, 14]. Moreover, a recent study by Liu et al. [15] investigated the prevalence of neurodevelopmental disorders in paediatric patients with intracranial germ cell tumours and found an association between ASD and pure germinomas.

Few studies have investigated the association between psychiatric disorders and TGCT. One previous study showed that patients with schizophrenia had a lower risk for testicular cancer [16].

The prognosis of TGCT is currently excellent, but some individuals present with a widely disseminated disease, requiring extensive treatment to be cured [4]. For other malignancies, patients with psychiatric disorders are more often diagnosed with advanced stage [17, 18]. Patients with severe psychiatric disorders may also have difficulties accepting cancer treatments [18]. One previous study of TGCT patients showed that a history of psychiatric disorders was associated with worse overall survival (OS) [19].

Our aim was to investigate if history of any neurodevelopmental or other psychiatric disorder is associated with increased risk of TGCT and increased mortality. We had two a priori hypotheses based on clinical observations. Firstly, we hypothesised that psychiatric disorders, specifically neurodevelopmental disorders such as ASD, ADHD and ID, are associated with an increased risk of developing TGCT. Secondly, we hypothesised that psychiatric disorders overall are associated with poorer TGCT prognosis.

## Materials and methods

### Study design

We first used a nested case–control study design to assess the risk of TGCT. We then used a cohort study design to investigate the associations between a history of psychiatric disorders and prognosis among the TGCT patients (i.e. among the cases in the case–control study).

### Study population

**Fig. 1:**
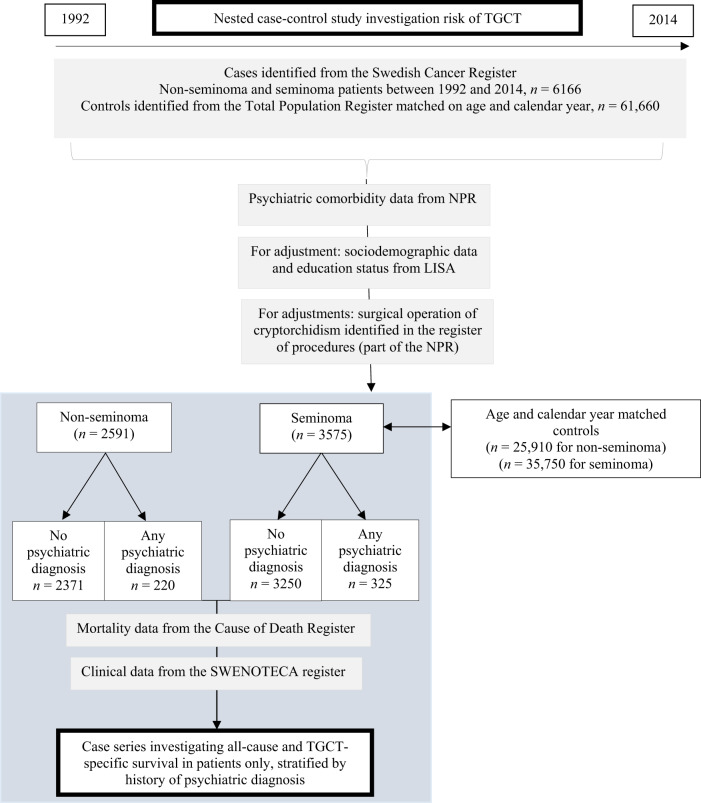
Flowchart describing data extraction from the Swedish registers and creation of the two cohorts under study. First, we have a case–control design for the aetiological results and a case series (grey box), for the survival results. The number of patients and controls are indicated, and also stratified by subtype and history of psychiatric comorbidity or not. TGCT testicular germ cell tumour, NPR National Patient Register, LISA Longitudinal Integrated Database for Health Insurance and Labour Market Studies, SWENOTECA Sweden Norway Testicular Cancer Group.

We included all patients diagnosed with TGCT in Sweden during the years 1992–2014 as cases (Fig. 1). The cases were identified from the Swedish Cancer Register using ICD-9 (186) and ICD-10 (C62), and ICD-O/3.2 (SNOMED/C24 codes, Table S1) and were individually matched for age and calendar period of diagnosis with 10 cancer-free controls each. The controls were identified from the Total Population Register. Mortality data were retrieved from the Swedish Cause of Death Register. TGCT-specific mortality was defined as death due to TGCT as a main or contributing cause of death.

### Exposures

Data on history of psychiatric diagnoses were retrieved from the nationwide Swedish Patient Register. The Swedish Patient Register includes all in-patient care, and since 2001 also outpatient physician consultations including psychiatric care from both public and private caregivers. History of any psychiatric disorder was defined as having any ICD9 diagnosis of 290–319 (1992–1996) or any ICD10 diagnosis of F00-F99 (after 1997). Diagnoses of psychiatric disorders were identified up until six months prior to the TGCT diagnosis for the cases and index date for the controls. The psychiatric disorders were stratified according to subtype into six categories: Psychotic disorders, Mood and anxiety disorders, Sleep disorders, Eating and personality disorders, Neurodevelopmental disorders and Substance misuse (Table S2).

Although a wide range of psychiatric disorders can be considered neurodevelopmental, we used the term neurodevelopmental disorders to describe this using DSM-5, i.e. ASD (299 or F84 according to ICD9 or ICD10, respectively), ADHD (314 or F90) and ID (317-319 or F70-79). According to DSM-5, communication disorders, specific learning disorders and motor disorders are also classified as neurodevelopmental disorders [20], but data on these disorders were not available.

### Covariates

Sociodemographic data were extracted from LISA, the Longitudinal Integrated Database for Health Insurance and Labour Market Studies at Statistics Sweden. The information was retrieved one year prior to the index year for the majority of cases and controls. Level of education was classified into three categories: 9 or less years of school, 10–12 years of school, or more than 12 years of school.

Clinical stage was defined according to Royal Marsden, modified [21]. Details of clinical stage were available from years 2000 and onwards and were identified from the Swedish Testicular Cancer Register held by the SWENOTECA-group. Patients in the study were treated according to the guidelines in the SWENOTECA protocols [22].

History of cryptorchidism was defined as having undergone a surgical correction and the information on operation of cryptorchidism was retrieved from the register of procedures, which is part of the National Patient Register. History of any unspecified congenital malformation was also retrieved from the National Patient Register (Table S2). Data on specific congenital malformations (e.g. hypospadias and unoperated cryptorchidism) was not available.

### Statistical analyses

We used conditional logistic regression (conditioned for age and calendar year) to estimate odds ratios (ORs) with 95% confidence intervals (CIs) for the association of psychiatric diagnoses with TGCT, overall and stratified by histopathological subgroup (seminoma and non-seminoma). We constructed a multivarible model adjusting for education, congenital malformations and cryptorchidism.

Thereafter, we used Kaplan–Meier failure curves and cumulative incidence functions to visualise the differences in mortality. Cox regression models were used to estimate hazard ratios (HRs) and 95% CI to assess the association between psychiatric comorbidity and all-cause and TGCT-specific mortality. We constructed two multivariable models, in the first model we adjusted for age, index year and education, the second model was further adjusted for cancer stage. In a sensitivity analysis educational level was left out of the model, and the results remained essentially unchanged. Due to limited statistical power, since there were few deaths, it was not possible to separately analyse mortality for different subtypes of psychiatric disorders. Follow-up started on the date of diagnosis, and ended on the date of death or administrative censoring (December 31, 2018) in all-cause mortality analyses. In analyses of TGCT-specific mortality, patients were followed from date of diagnosis until death with the underlying cause of death recorded as TGCT as the outcome event, with censoring at death due to other causes, or to administrative end of follow-up (December 31, 2017). Statistical analyses were performed using the SAS 9.4 (SAS Institute, Cary, NC) software.

## Results

We included 6166 TGCT patients, of whom 2591 were diagnosed with non-seminoma and 3575 with seminoma, and 61,660 matched controls (Table 1). As expected, history of cryptorchidism was more prevalent among patients than controls.

**Table 1:** Characteristics of cancer patients in subgroups of patients with non-seminoma and seminoma and the matched general population controls.

Variable	Non-seminoma			Seminoma			Overall		
	Cases	Controls	*p* value	Cases	Controls	*p* value	Cases	Controls	*p* value
*N*	2591	25,910		3575	35,750		6166	61,660	
*Age*									
Median (IQR)	29 (24–36)	29 (24–36)	1.0	38 (32–46)	38 (32–46)	1.0	34 (28–42)	34 (28–42)	1.0
Range, min–max	18–78	18–78	–	18–79	18–79	–	18–79	18–79	
Categories									
18–29 years	1323 (51.1)	13,230 (51.1)	1.0	636 (17.8)	6360 (17.8)	1.0	1959 (31.8)	19,590 (31.8)	1.0
30–39 years	824 (31.8)	8240 (31.8)		1401 (39.2)	14,010 (39.2)		2225 (36.1)	22,250 (36.1)	
40–49 years	285 (11.0)	2850 (11.0)		920 (25.7)	9200 (25.7)		1205 (19.5)	12,050 (19.5)	
≥50 years	159 (6.1)	1590 (6.1)		618 (17.3)	6180 (17.3)		777 (12.6)	7770 (12.6)	
*Index year*									
1992–2000	855 (33.0)	8550 (33.0)	1.0	1143 (32.0)	11,430 (32.0)	1.0	1998 (32.4)	19,980 (32.4)	1.0
2001–2008	934 (36.0)	9340 (36.0)		1267 (35.4)	12,670 (35.4)		2201 (35.7)	22,010 (35.7)	
2009–2014	802 (31.0)	8020 (31.0)		1165 (32.6)	11,650 (32.6)		1967 (31.9)	19,670 (31.9)	
*Education*^a^									
<9 years	494 (19.1)	4559 (17.6)	0.02	580 (16.2)	6494 (18.2)	0.006	1074 (17.4)	11,053 (17.9)	0.05
10–12 years	1371 (52.9)	13,916 (53.7)		1812 (50.7)	18,226 (51.0)		3183 (51.6)	32,142 (52.1)	
≥12 years	672 (25.9)	7054 (27.2)		1138 (31.8)	10,651 (29.8)		1810 (29.4)	17,705 (28.7)	
Missing	54 (2.1)	381 (1.5)		45 (1.3)	379 (1.1)		99 (1.6)	760 (1.2)	
*Swedish background*^b^									
Yes	2240 (86.5)	21,983 (84.8)	<0.001	3 152 (88.2)	30,204 (84.5)	<0.001	5 392 (87.4)	52,187 (84.6)	<0.001
No	345 (13.3)	3925 (15.1)		422 (11.8)	5543 (15.5)		767 (12.4)	9468 (15.4)	
Missing	6 (0.2)	2 (0.0)		1 (0.0)	3 (0.0)		7 (0.1)	5 (0.0)	
*Clinical stage*									
CS I	1280 (49.4)			2123 (59.4)			3403 (55.2)		
CS II	483 (18.6)			280 (7.8)			763 (12.4)		
CS III	47 (1.8)			35 (1.0)			82 (1.3)		
CS IV	375 (14.5)			37 (1.0)			412 (6.7)		
CS Mk+	70 (2.7)			2 (0.1)			72 (1.2)		
Missing	336 (13.0)			1098 (30.7)			1434 (23.3)		
Missing <2000	302 (11.7)			969 (27.1)			1271 (20.6)		
Malformation and cryptorchidism									
Any malformation	74 (2.9)	611 (2.4)	0.11	90 (2.5)	621 (1.7)	<0.001	164 (2.7)	1 232 (2.0)	<0.001
Cryptorchidism	66 (2.5)	294 (1.1)	<0.001	73 (2.0)	355 (1.0)	<0.001	139 (2.3)	649 (1.1)	<0.001
Any malformation (ICD) or cryptorchidism	122 (4.7)	810 (3.1)	<0.001	149 (4.2)	887 (2.5)	<0.001	271 (4.4)	1 697 (2.8)	<0.001

Data are presented as numbers (%) unless otherwise specified.

*IQR* interquartile range, *CS* clinical stage, *Mk+* marker positive.

^a^In years of schooling.

^b^Swedish background defined as born in Sweden with at least one parent born in Sweden.

### Risk of TGCT in patients with psychiatric disorders

A history of any psychiatric disorder overall was present in 8.8% of the patients and 8.3% of the controls indicating no significant increased risk of TGCT (OR 1.06; 95% CI 0.96–1.17, Table 2). Comparing TGCT overall to controls revealed no statistically significant differences in proportions of psychiatric disorders. This was also the case for the subgroup of non-seminoma patients.

**Table 2:** Odds ratios for the risk of testicular germ cell tumour by history of psychiatric disorders.

Exposure	*N* subjects (% exposed)		Model^a^	
	Cases	Controls	Odds ratio (95% CI)	*p* value
*Overall*				
Any psychiatric diagnosis	545 (8.8)	5132 (8.3)	1.06 (0.96–1.17)	0.22
Psychotic disorders	45 (0.7)	529 (0.9)	0.85 (0.62–1.15)	0.29
Mood and anxiety disorders	214 (3.5)	2282 (3.7)	0.93 (0.80–1.07)	0.32
Sleep disorders	9 (0.1)	88 (0.1)	1.04 (0.52–2.07)	0.91
Eating and personality disorders	42 (0.7)	416 (0.7)	1.01 (0.74–1.39)	0.94
Neurodevelopmental disorders	70 (1.1)	547 (0.9)	1.21 (0.93–1.56)	0.16
Intellectual disabilities	24 (0.4)	173 (0.3)	1.13 (0.72–1.77)	0.58
ADHD	32 (0.5)	252 (0.4)	1.27 (0.88–1.85)	0.20
ASD	21 (0.3)	204 (0.3)	0.97 (0.62–1.54)	0.91
ADHD or ASD	48 (0.8)	413 (0.7)	1.14 (0.84–1.55)	0.39
Substance misuse	202 (3.3)	1924 (3.1)	1.07 (0.92–1.24)	0.39
*Non-seminoma*				
Any psychiatric diagnosis	220 (8.5)	2066 (8.0)	1.02 (0.88–1.18)	0.79
Psychotic disorders	24 (0.9)	181 (0.7)	1.25 (0.82–1.93)	0.30
Mood and anxiety disorders	81 (3.1)	904 (3.5)	0.86 (0.68–1.09)	0.22
Sleep disorders	3 (0.1)	35 (0.1)	0.85 (0.26–2.77)	0.79
Eating and personality disorders	17 (0.7)	172 (0.7)	0.94 (0.57–1.56)	0.82
Neurodevelopmental disorders	31 (1.2)	295 (1.1)	0.91 (0.62–1.34)	0.64
Intellectual disabilities	12 (0.5)	91 (0.4)	0.99 (0.53–1.87)	0.99
ADHD	16 (0.6)	139 (0.5)	1.08 (0.64–1.82)	0.79
ASD	8 (0.3)	115 (0.4)	0.60 (0.29–1.24)	0.17
ADHD or ASD	21 (0.8)	226 (0.9)	0.84 (0.53–1.33)	0.47
Substance misuse	86 (3.3)	752 (2.9)	1.11 (0.88–1.40)	0.36
*Seminoma*				
Any psychiatric diagnosis	325 (9.1)	3066 (8.6)	1.08 (0.96–1.22)	0.20
Psychotic disorders	**21 (0.6)**	**348 (1.0)**	**0.62 (0.40–0.96)**	**0.034**
Mood and anxiety disorders	133 (3.7)	1378 (3.9)	0.97 (0.81–1.17)	0.75
Sleep disorders	6 (0.2)	53 (0.1)	1.17 (0.50–2.73)	0.71
Eating and personality disorders	25 (0.7)	244 (0.7)	1.06 (0.70–1.60)	0.79
Neurodevelopmental disorders	**39 (1.1)**	**252 (0.7)**	**1.54 (1.09–2.19)**	**0.015**
Intellectual disabilities	12 (0.3)	82 (0.2)	1.28 (0.68–2.42)	0.45
ADHD	16 (0.4)	113 (0.3)	1.47 (0.87–2.49)	0.15
ASD	13 (0.4)	89 (0.2)	1.50 (0.83–2.71)	0.18
ADHD or ASD	27 (0.8)	187 (0.5)	1.50 (0.99–2.26)	0.05
Substance misuse	116 (3.2)	1172 (3.3)	1.03 (0.85–1.25)	0.76

*ADHD* attention deficit hyperactivity disorders, *ASD* autism spectrum disorders.

Significant associations are indicated in bold.

^a^Model: conditioned on matching set (age and calendar year) and further adjusted for education, and congenital malformation or surgery for cryptorchidism.

However, neurodevelopmental disorders were seen among 1.1% of the seminoma patients and among 0.7% of their controls and psychotic disorders were seen among 0.6% of the seminoma patients and among 1.0% of their controls. History of a neurodevelopmental disorder was associated with an increased risk of seminoma (OR 1.54; 95% CI 1.09–2.19) whereas a history of a psychotic disorder was associated with a decreased risk of seminoma (OR 0.62; 95% CI 0.40–0.96) (Table 2).

### Age at diagnosis

Median age at diagnosis was 29 years for non-seminoma patients overall and 23 years for non-seminoma patients with neurodevelopmental disorders (Wilcoxon–Mann–Whitney test *p* < 0.001, Table S3). Median age at diagnosis was 38 years for seminoma patients overall and 34 years for seminoma patients with neurodevelopmental disorders (*p* = 0.004, Table S4).

### Stage at diagnosis

Stage IV cancer was seen in 5.4% of the seminoma patients with a neurodevelopmental disorder and in 1.2% of those with no psychiatric disorders (Table S4). A significant difference in stage distribution overall was noted for seminoma patients (Fisher’s exact test *p* = 0.036), but not for non-seminoma patients (Fisher’s exact test *p* = 0.264).

### Mortality

**Fig. 2:**
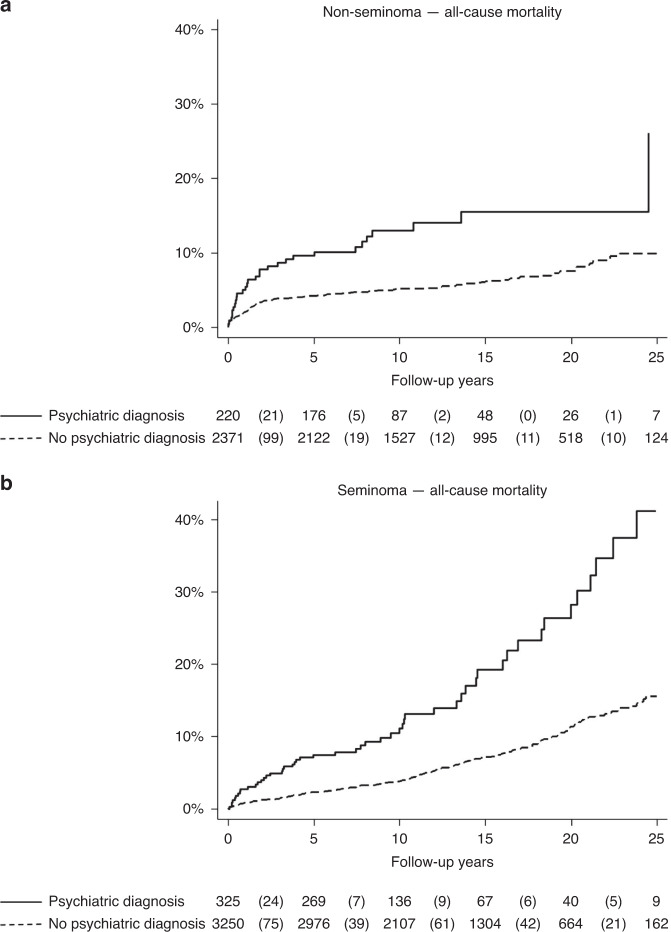
All-cause mortality in patients with and without prior psychiatric disorders. Kaplan–Meier failure curves for all-cause mortality in patients with non-seminoma (**a**) and seminoma (**b**) with psychiatric diagnosis (solid line) and without history of psychiatric diagnosis (dashed line).

The all-cause mortality among patients with seminoma and non-seminoma was increased for patients with a history of any psychiatric disorder compared to patients with no history of psychiatric disorders (Fig. 2). This persisted when adjusted for age, index year, education and when further adjusted for cancer stage both in patients overall (HR 2.91; 95% CI 2.11–4.02), and when stratified by subgroup in non-seminoma patients and seminoma patients (Table 3).

**Table 3:** Risk of all-cause mortality and testicular germ cell tumour-specific mortality overall and by subgroups with and without history of psychiatric diagnosis (hazard ratios with 95% confidence intervals).

Group	*N* events/*N* (%)		Mortality rate (95% CI) per 1000 PY		Model I HR (95% CI)	Model II HR (95% CI)
	Psychiatric diagnosis	No psychiatric diagnosis	Psychiatric diagnosis	No psychiatric diagnosis		
All-cause mortality						
Overall	80/545 (14.7%)	393/5621 (7.0%)	4.2 (3.3–5.1)	2.1 (1.8–2.3)	**2.21 (1.71–2.85)**	**2.91 (2.11–4.02)**
Non-seminoma	29/220 (13.2%)	151/2371 (6.4%)	4.3 (2.7–5.9)	2.1 (1.8–2.5)	**1.99 (1.31–3.03)**	**2.57 (1.60–4.11)**
Seminoma	51/325 (15.7%)	242/3250 (7.4%)	4.1 (3.0–5.3)	2.0 (1.7–2.2)	**2.63 (1.90–3.64)**	**3.33 (2.10–5.29)**
TGCC-specific mortality						
Overall	24/545 (4.4%)	133/5621 (2.4%)	4.6 (2.8–6.5)	1.9 (1.5–2.2)	**1.76 (1.13–2.76)**	**1.79 (1.04–3.08)**
Non-seminoma	14/220 (6.4%)	87/2371 (3.7%)	6.8 (3.2–10.3)	2.9 (2.3–3.5)	1.65 (0.92–2.96)	1.50 (0.77–2.94)
Seminoma	10/325 (3.1%)	46/3250 (1.4%)	3.2 (1.2–5.2)	1.1 (0.8–1.4)	**2.76 (1.36–5.60)**	**3.12 (1.19–8.17)**

Model I: Adjusted for age, index year, and education; Model II: Model I and further adjusted for cancer stage (CS I, CS II + CS Mk+, CS III + CS IV).

Significant associations are indicated in bold.

*HR* hazard ratios, *PY* person years.

**Fig. 3:**
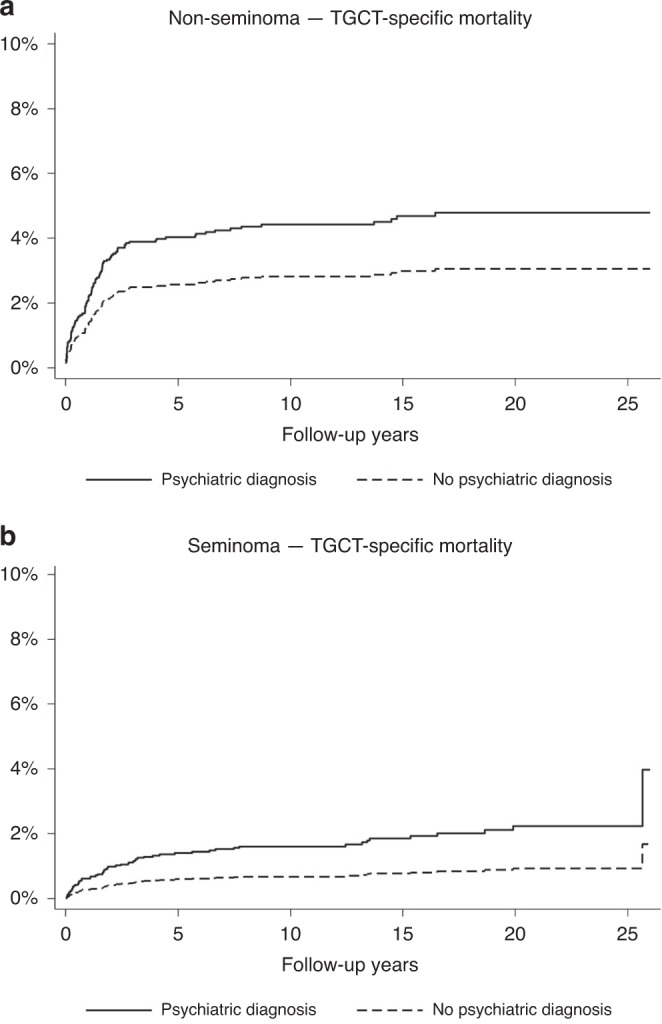
Testicular germ cell tumour-specific mortality in patients with and without prior psychiatric disorders. Cumulative incidence function using non-testicular cancer mortality as competing event for cause-specific testicular cancer mortality in patients with (**a**) non-seminoma and (**b**) seminoma with and without history of psychiatric diagnosis.

Furthermore, the TGCT-specific mortality was increased among patients overall with history of any psychiatric disorder (HR 1.79; 95% CI 1.04–3.08). Stratified by subgroup, the TGCT-specific mortality was increased among seminoma patients with a history of any psychiatric disorder (HR 3.12; 95% CI 1.19–8.17, adjusted for stage), but not among non-seminoma patients with such history (Table 3 and Fig. 3).

### Sensitivity analyses

Since the Swedish Patient Register also started to record outpatient visits from year 2001 and onwards, stratified analyses by calendar period were conducted (Table S5). The association between seminoma and neurodevelopmental disorders remained when excluding patients before 2001 (HR 1.52 95% CI 1.06–2.19) but the inverse association between seminoma and psychotic disorders was no longer significant (HR 0.69 95% CI 0.42–1.13) (Table S6). The all-cause and TGCT-specific mortality remained significantly increased in analyses restricted to index year ≥2001 (Overall HR 2.83 95% CI 1.98–4.05 and HR 2.12 95% CI 1.19–3.77, respectively) (Table S7).

## Discussion

Having a history of a neurodevelopmental disorder, such as ASD, ADHD and ID, was associated with an increased risk of seminoma whereas a history of a psychotic disorder was associated with a decreased risk of seminoma. In addition, in comparison to patients without psychiatric comorbidity, both the all-cause mortality and the TGCT-specific mortality were increased for patients with a history of psychiatric disorders. Due to insufficient number of patients, it was not possible to separately analyse mortality in the group of patients with neurodevelopmental disorders. We found no association between history of psychiatric disorders, including neurodevelopmental disorders, and risk of TGCT overall.

Only one previous study has investigated the prevalence of neurodevelopmental disorders in patients with germ cell tumours, and this study investigated paediatric patients with intracranial germ cell tumours (*n* = 111). They found an ASD prevalence among patients with pure germinoma that was estimated to be more than threefold higher than the national prevalence estimate [15]. This discrepancy in ASD prevalence was not seen among patients with intracranial non-germinomatous germ cell tumours. Since intracranial pure germinomas are histologically identical to seminomas of the testis [23], this accords with our results positing an association between seminomas and neurodevelopmental disorders. The ADHD prevalence among both pure germinoma and non-germinomatous germ cell tumours, was in line with the current estimated ADHD prevalence rate.

Furthermore, in a recent study by Liu Q et al. [24] the risk of testicular cancer among patients with ASD was slightly, but not significantly, increased (OR 1.4; 95% CI 0.8–2.3). However, they did not study other neurodevelopmental disorders, adjust for malformations or present results for seminoma and non-seminoma patients separately.

Previous studies have found associations between TGCT and urogenital malformations [25, 26] and between urogenital malformations and neurodevelopmental disorders such as ASD and ADHD [11, 12]. Since our results between neurodevelopmental disorders and seminoma remained when adjusted for cryptorchidism and congenital malformations, this could indicate that there are other possible shared risk factors than these, or a shared susceptibility between TGCT and neurodevelopmental disorders. Interestingly, seminoma patients with neurodevelopmental disorders in our study were diagnosed at an earlier age, and at a more advanced stage, compared to patients without such comorbidity, potentially indicating an earlier penetrance of the disease in these patients.

Neurodevelopmental disorders, such as ASD, ADHD and ID, are complex and multifactorial and can be a result of both genetic and environmental factors [13, 27, 28]. There is support for these diagnoses to be grouped together due to their clinical course, prominent early onset neurocognitive deficits, their typically multifactorial origin and the high level of overlap between these disorders and their constituent symptom dimensions [20]. Possible shared risk factors between these disorders and TGCT could be genetic, and there are studies that have found an overlap in risk genes for ASD and for cancer. Altered regulation of the RAS/MAPK and PI3K/AKT/mTOR pathways are e.g. associated with autism and other neurodevelopmental disorders, and with the development of cancer [29–31] and alterations in these pathways are also involved in the development of TGCT [32, 33]. Furthermore, alterations in oestrogen and/or androgen levels in the in-utero environment are thought to be a factor in both the development of TGCT [34] and in influencing the development of the nervous system [35]. A connection between endocrine disruptors and TGCT [36, 37], and similarly an association between endocrine disruptors and neurodevelopmental disorders [38] has been suggested. Both the incidence of TGCT and the incidence of neurodevelopmental disorders are increasing, and there are theories that this could partly be due to exposure to endocrine disruptors [36–38].

We also found an inverse association between seminoma and patient history of psychotic disorders. Similarly, Mortensen et al. showed that a lower risk of TGCT was seen among patients with schizophrenia [16]. Although there are studies that show a lower risk of several types of cancer among patients with schizophrenia [39, 40], few other studies have investigated the risk of TGCT specifically. One study by Ji et al. [40] found a generally lower cancer risk among patients with schizophrenia, but not among their TGCT patients. There are hypotheses regarding a genetic cause of the decreased risk of cancer among these patients [39, 41], for example, reduced activation of the above mentioned PI3K/AKT/mTOR pathway seems to be associated with schizophrenia [30, 42]. Another hypothesis has been that antipsychotic drugs could have a protective effect [43].

Somewhat unexpectedly, we only found statistically significant results for seminoma and not for non-seminoma patients. Although the causes of seminoma and non-seminoma are largely considered to be the same [44, 45], there are studies that suggest that some risk factors are more strongly associated with seminoma or non-seminoma [44], which may provide part of the explanation to our findings.

It has previously been reported that patients with psychiatric disorders have an increased all-cause mortality compared to the general population [46]. In the only study we could find investigating the prognosis among TGCT-patients with a history of psychiatric disorders, Kishimoto et al. [19] reported a decreased OS among TGCT-patients with a history of severe psychiatric disorders, which is consistent with our results. Kishimoto et al. did not find significantly decreased cancer-specific survival among patients with severe psychiatric disorders, however, they did not study seminoma and non-seminoma separately. Moreover, their study included only patients with severe psychiatric disorders, and consequently did not include patients with, e.g. neurodevelopmental disorders.

Patients with psychiatric disorders tend to seek medical attention later than other patients [17, 18]. Thus, the stage distribution could contribute to the worse prognosis among these patients in our study. However, this cannot be the sole explanation since our findings of increased TGCT-specific mortality among patients with psychiatric disorders remained even when adjusted for stage. Another possible explanation to the worse prognosis among these patients could be difficulties in taking part in cancer treatment.

In a recent study by Chang et al. [47], testicular cancer patients had the highest cumulative burden of psychiatric disorders across all 26 researched adult cancers. The high cumulative burden of psychiatric disorders, along with the worse prognosis among TGCT patients with psychiatric disorders, adds to the importance of investigating this further, in future studies.

Strengths of this study were the nationwide scope including nearly all cases of TGCT in Sweden during the time period, and the availability of clinical data through the SWENOTECA register. We were also able to identify all patients that were diagnosed with psychiatric disorders through prospectively recorded registrations, thus eliminating differential misclassification bias that may otherwise be present in case-control studies.

Studies show an increasing prevalence of ADHD and ASD in Sweden during the period of this study [48, 49]. Though it is possible that some of this increase is due to a true increase in prevalence, most studies argue that the rise in prevalence may at least partly be due to an increased awareness and/or changes in diagnostic practices. This could mean that there are patients in our cohort that have not yet been diagnosed with a neurodevelopmental disorder. This could possibly also explain the lower age at TGCT diagnosis among patients with neurodevelopmental disorders. Patients with younger age at TGCT diagnosis are more likely to have been diagnosed with a neurodevelopmental disorder since the frequency of neurodevelopmental disorders have increased over time. Additionally, the Swedish Patient Register does not cover outpatient visits from before 2001 or primary care. Patients in our cohort with less severe psychiatric disorders that have not required inpatient or specialised care may therefore in some cases be unidentified. However, when excluding all patients with index year before 2001, our main results were not altered, except for the inverse association between psychotic disorders and seminoma. Nevertheless, this could be due to a decrease in power in the analysis.

To exclude psychiatric diagnoses caused by TGCT, e.g. stress reactions on receiving the diagnosis, we excluded psychiatric disorders registered within six months prior to the cancer diagnosis. Apart from this, all psychiatric disorders in these patients were included. This may be a strength in our study, but also a limitation since we may have included diagnoses that have later been removed.

## Conclusion

To our knowledge, this is the first study that has investigated and observed an association between neurodevelopmental disorders and testicular seminoma. This finding would in part confirm clinical observations suggesting an association between neurodevelopmental disorders and TGCT. However, as the difference in risk was relatively small, this may not be clinically relevant, and moreover, we cannot exclude the possibility that this finding was due to chance. Hence, our finding would have to be replicated in other studies in order to be confirmed.

Furthermore, we found an increased TGCT-specific mortality for TGCT patients, and in particular for seminoma patients with psychiatric disorders, compared to patients without psychiatric disorders. This is important to keep in mind, suggesting that one may need to be extra observant in providing these patients with the same opportunities to being successively treated, cured and followed for their TGCT.

## Supplementary information

Appendix_Psychiatric disorders and TGCT

## Data Availability

The data in our study result from linkages of nationwide registers as described in the method section. Restrictions apply for the availability of these data according to the national data protection legislation. Data are available from the authors with the permission of the Swedish Authority for Privacy Protection. Additional information will be available from the corresponding author upon request.

## References

[1] Sung H, Ferlay J, Siegel RL, Laversanne M, Soerjomataram I, Jemal A (2021). Global Cancer Statistics 2020: GLOBOCAN estimates of incidence and mortality worldwide for 36 cancers in 185 countries. CA Cancer J Clin.

[2] Znaor A, Skakkebaek NE, Rajpert-De Meyts E, Laversanne M, Kuliš T, Gurney J (2020). Testicular cancer incidence predictions in Europe 2010-2035: a rising burden despite population ageing. Int J Cancer.

[3] Gurney JK, Florio AA, Znaor A, Ferlay J, Laversanne M, Sarfati D (2019). International trends in the incidence of testicular cancer: lessons from 35 years and 41 countries. Eur Urol.

[4] Rajpert-De Meyts E, McGlynn KA, Okamoto K, Jewett MA, Bokemeyer C (2016). Testicular germ cell tumours. Lancet..

[5] Pluta J, Pyle LC, Nead KT, Wilf R, Li M, Mitra N (2021). Identification of 22 susceptibility loci associated with testicular germ cell tumors. Nat Commun.

[6] Cook MB, Akre O, Forman D, Madigan MP, Richiardi L, McGlynn KA (2010). A systematic review and meta-analysis of perinatal variables in relation to the risk of testicular cancer-experiences of the son. Int J Epidemiol.

[7] McGlynn KA, Trabert B (2012). Adolescent and adult risk factors for testicular cancer. Nat Rev Urol.

[8] Ghasemiesfe M, Barrow B, Leonard S, Keyhani S, Korenstein D (2019). Association between marijuana use and risk of cancer: a systematic review and meta-analysis. JAMA Netw Open.

[9] Skakkebaek NE, Rajpert-De Meyts E, Main KM (2001). Testicular dysgenesis syndrome: an increasingly common developmental disorder with environmental aspects. Hum Reprod.

[10] Hoei-Hansen CE, Rajpert-De Meyts E, Daugaard G, Skakkebaek NE (2005). Carcinoma in situ testis, the progenitor of testicular germ cell tumours: a clinical review. Ann Oncol.

[11] Chen J, Sørensen HT, Miao M, Liang H, Ehrenstein V, Wang Z (2018). Cryptorchidism and increased risk of neurodevelopmental disorders. J Psychiatr Res.

[12] Butwicka A, Lichtenstein P, Landén M, Nordenvall AS, Nordenström A, Nordenskjöld A (2015). Hypospadias and increased risk for neurodevelopmental disorders. J Child Psychol Psychiatry.

[13] Huang J, Zhu T, Qu Y, Mu D (2016). Prenatal, perinatal and neonatal risk factors for intellectual disability: a systemic review and meta-analysis. PLoS ONE.

[14] Carlsson T, Molander F, Taylor MJ, Jonsson U, Bölte S. Early environmental risk factors for neurodevelopmental disorders - a systematic review of twin and sibling studies. Dev Psychopathol. 2021:33:1448–95. 10.1017/S0954579420000620 PMC8564717 32703331

[15] Liu KX, Sethi RV, Pulsifer MB, D’Gama AM, LaVally B, Ebb DH (2021). Clinical outcomes of pediatric patients with autism spectrum disorder and other neurodevelopmental disorders and intracranial germ cell tumors. Pediatr Blood Cancer.

[16] Mortensen PB (1994). The occurrence of cancer in first admitted schizophrenic patients. Schizophr Res.

[17] Kaerlev L, Iachina M, Trosko O, Qvist N, Ljungdalh PM, Nørgård BM (2018). Colon cancer patients with a serious psychiatric disorder present with a more advanced cancer stage and receive less adjuvant chemotherapy - a nationwide Danish cohort study. BMC Cancer.

[18] Shinden Y, Kijima Y, Hirata M, Nakajo A, Tanoue K, Arigami T (2017). Clinical characteristics of breast cancer patients with mental disorders. Breast..

[19] Kishimoto N, Takao T, Tsutahara K, Tanigawa G, Yamaguchi S (2016). Clinical analysis of severe psychiatric disorders in patients with testicular cancer: a single-center experience. Int J Urol.

[20] Thapar A, Cooper M, Rutter M (2017). Neurodevelopmental disorders. Lancet Psychiatry.

[21] Peckham MJ, McElwain TJ, Barrett A, Hendry WF (1979). Combined management of malignant teratoma of the testis. Lancet..

[22] Tandstad T, Ståhl O, Håkansson U, Wahlqvist R, Klepp O, Cavallin-Ståhl E (2016). The SWENOTECA group: a good example of continuous binational and multidisciplinary collaboration for patients with testicular cancer in Sweden and Norway. Scand J Urol..

[23] Plant AS, Chi SN, Frazier L (2016). Pediatric malignant germ cell tumors: a comparison of the neuro-oncology and solid tumor experience. Pediatr Blood Cancer.

[24] Liu Q, Yin W, Meijsen JJ, Reichenberg A, Gådin JR, Schork AJ (2022). Cancer risk in individuals with autism spectrum disorder. Ann Oncol.

[25] Trabert B, Zugna D, Richiardi L, McGlynn KA, Akre O (2013). Congenital malformations and testicular germ cell tumors. Int J Cancer.

[26] Daltveit DS, Klungsøyr K, Engeland A, Ekbom A, Gissler M, Glimelius I (2020). Cancer risk in individuals with major birth defects: large Nordic population based case-control study among children, adolescents, and adults. BMJ..

[27] Thapar A, Cooper M (2016). Attention deficit hyperactivity disorder. Lancet..

[28] Chaste P, Leboyer M (2012). Autism risk factors: genes, environment, and gene-environment interactions. Dialog Clin Neurosci.

[29] Crawley JN, Heyer WD, LaSalle JM (2016). Autism and cancer share risk genes, pathways, and drug targets. Trends Genet.

[30] Crespi B (2011). Autism and cancer risk. Autism Res.

[31] Borrie SC, Brems H, Legius E, Bagni C (2017). Cognitive dysfunctions in intellectual disabilities: the contributions of the Ras-MAPK and PI3K-AKT-mTOR pathways. Annu Rev Genomics Hum Genet.

[32] Ichimura K, Fukushima S, Totoki Y, Matsushita Y, Otsuka A, Tomiyama A (2016). Recurrent neomorphic mutations of MTOR in central nervous system and testicular germ cell tumors may be targeted for therapy. Acta Neuropathol.

[33] McIntyre A, Gilbert D, Goddard N, Looijenga L, Shipley J (2008). Genes, chromosomes and the development of testicular germ cell tumors of adolescents and adults. Genes Chromosomes Cancer.

[34] Giannandrea F, Paoli D, Figà-Talamanca I, Lombardo F, Lenzi A, Gandini L (2013). Effect of endogenous and exogenous hormones on testicular cancer: the epidemiological evidence. Int J Dev Biol.

[35] Romano E, Cosentino L, Laviola G, De Filippis B (2016). Genes and sex hormones interaction in neurodevelopmental disorders. Neurosci Biobehav Rev.

[36] Lymperi S, Giwercman A (2018). Endocrine disruptors and testicular function. Metabolism..

[37] Bräuner EV, Lim YH, Koch T, Uldbjerg CS, Gregersen LS, Pedersen MK (2021). Endocrine disrupting chemicals and risk of testicular cancer a systematic review and meta-analysis. J Clin Endocrinol Metab.

[38] Rivollier F, Krebs MO, Kebir O (2019). Perinatal exposure to environmental endocrine disruptors in the emergence of neurodevelopmental psychiatric diseases: a systematic review. Int J Environ Res Public Health.

[39] Tabarés-Seisdedos R, Dumont N, Baudot A, Valderas JM, Climent J, Valencia A (2011). No paradox, no progress: inverse cancer comorbidity in people with other complex diseases. Lancet Oncol.

[40] Ji J, Sundquist K, Ning Y, Kendler KS, Sundquist J, Chen X (2013). Incidence of cancer in patients with schizophrenia and their first-degree relatives: a population-based study in Sweden. Schizophr Bull.

[41] Tabarés-Seisdedos R, Rubenstein JL (2009). Chromosome 8p as a potential hub for developmental neuropsychiatric disorders: implications for schizophrenia, autism and cancer. Mol Psychiatry.

[42] Enriquez-Barreto L, Morales M (2016). The PI3K signaling pathway as a pharmacological target in autism related disorders and schizophrenia. Mol Cell Ther.

[43] Li H, Li J, Yu X, Zheng H, Sun X, Lu Y (2018). The incidence rate of cancer in patients with schizophrenia: a meta-analysis of cohort studies. Schizophr Res.

[44] McGlynn KA, Cook MB (2009). Etiologic factors in testicular germ-cell tumors. Future Oncol.

[45] Bray F, Richiardi L, Ekbom A, Forman D, Pukkala E, Cuninkova M (2006). Do testicular seminoma and nonseminoma share the same etiology? Evidence from an age-period-cohort analysis of incidence trends in eight European countries. Cancer Epidemiol Biomark Prev.

[46] Chesney E, Goodwin GM, Fazel S (2014). Risks of all-cause and suicide mortality in mental disorders: a meta-review. World Psychiatry.

[47] Chang WH, Lai AG (2022). Cumulative burden of psychiatric disorders and self-harm across 26 adult cancers. Nat Med.

[48] Giacobini M, Medin E, Ahnemark E, Russo LJ, Carlqvist P (2018). Prevalence, patient characteristics, and pharmacological treatment of children, adolescents, and adults diagnosed with ADHD in Sweden. J Atten Disord.

[49] Idring S, Lundberg M, Sturm H, Dalman C, Gumpert C, Rai D (2015). Changes in prevalence of autism spectrum disorders in 2001-2011: findings from the Stockholm youth cohort. J Autism Dev Disord.

