# Supplementary material for: Risk and mortality of testicular cancer in patients with neurodevelopmental or other psychiatric disorders
Source: Br J Cancer. 2023 Apr 24;128(12):2261–9. doi: 10.1038/s41416-023-02260-8 (PMC10241835; doi:10.1038/s41416-023-02260-8)
Supplement: Supplementary file 1 — Appendix_Psychiatric disorders and TGCT [file 41416_2023_2260_MOESM1_ESM.docx]

# APPENDIX

**Table S1** Definition of subgroups using the Cancer register

| **Variable** | **PAD** | **SNOMED** | **ICD10**  **(≥1997)** | **ICD9**  **(1987-1996)** |
| --- | --- | --- | --- | --- |
| **Testicular Germ Cell Cancer subtype** |  |  | C62 | 186 |
| Non-seminoma | 826, 806, 064, 823 | 90612, 906130, 90642, 90653, 90703, 90713, 90801, 90803, 90853, 91003, 91013, 90643, 90723, 90813, 91003 |  |  |
| Seminoma | 066 | 90613, 90633* |  |  |

*Spermatocytic tumors could not be distinguished from TGCC in the whole study period of 1992-2014 and were therefore included in the analysis. Detailed morphology codes of spematocytic tumors were available from year 2005 (SNOMED3/ICD-O/3). 28 patients with spermatocytic tumors (SNOMED3 code 90633) were identified. When spermatocytic tumors were excluded from the seminoma group the association between seminoma and neurodevelopmental disorders remained significant (HR 1.55; 95% CI 1.09-2.0), as did the inverse association with psychotic disorders (HR 0.62 95% CI 0.40-0.97) (data not shown).

**Table S2** Definition of exposures using ICD9-ICD10 codes and surgery codes

| **Variable** | **ICD10**  **(≥1997)** | **ICD9**  **(1987-1996)** | **Surgery codes**  **≥1997** | **Surgery codes**  **<1997** |
| --- | --- | --- | --- | --- |
|  |  |  |  |  |
| **Any psychiatric disease** | **F00-F99** | **290-319** |  |  |
| Psychotic disorders | F20-29 | 295, 297−299 (except 298A) |  |  |
| Mood-and anxiety disorders | F30-43 | 296A, 296C−E, 296W, 296X, 298A, 300, 302, 305B, 307A−H, 307X, 308- 311 |  |  |
| Sleep disorders | F51 | 307E |  |  |
| Eating disorders and personality disorders | F50, F60-F69 | 307B, 307F, 301 |  |  |
| Neurodevelopmental disorders | F70-79, F84, F90 | 317−319, 299, 314 |  |  |
| Intellectual disabilities | F70-79 | 317-319 |  |  |
| ADHD * | F90 | 314 |  |  |
| ASD** | F84 | 299 |  |  |
| ADHD or ASD | F84, F90 | 299, 314 |  |  |
| Substance misuse | F10-F19 | 291, 292, 303, 304, 305A, 305X |  |  |
|  |  |  |  |  |
| Any malformation | Q00-Q99 | 740-759, 771A-771C, 228A-228B, 279L, 524 |  |  |
| Cryptorchidism |  |  | KFH00, KFH10 | 6790 |

Abbreviations: ADHD, Attention deficit activity disorders; ASD, Autism spectrum disorders; ADD, Attention deficit disorder; DAMP, Deficits in attention, motor and perception

*ADHD includes: ADHD, ADD, Hyperactivity, DAMP

**ASD disorders include: Asperger’s syndrome, autism, other pervasive developmental disorders, Retts syndrome

**Table S3** Clinical stage **in non-seminoma patients** with and without history of psychiatric diagnosis

| **Variable** | **No psychiatric diagnosis** | **Any psychiatric diagnosis** | **Neurodevelopmental disorders** |
| --- | --- | --- | --- |
| N | 2 371 | 220 | 31 |
| **Age** |  |  |  |
| Median (IQR) | 29 (24-36) | 29 (24-37) | 23 (20-29) |
| Range, min-max | 18-78 | 18-71 | 18-71 |
| *Categories* |  |  |  |
| 18-29 years | 1 209 (51.0) | 114 (51.8) | 24 (77.4) |
| 30-39 years | 756 (31.9) | 68 (30.9) | 5 (16.1) |
| 40-49 years | 261 (11.0) | 24 (10.9) | 1 (3.2) |
| ≥50 years | 145 (6.1) | 14 (6.4) | 1 (3.2) |
| **Index year** |  |  |  |
| 1992-2000 | 815 (34.4) | 40 (18.2) | 2 (6.5) |
| 2001-2008 | 868 (36.6) | 66 (30.0) | 6 (19.4) |
| 2009-2014 | 688 (29.0) | 114 (51.8) | 23 (74.2) |
| **Education*** |  |  |  |
| <9 years | 414 (17.5) | 80 (36.4) | 15 (48.4) |
| 10-12 years | 1 271 (53.6) | 100 (45.5) | 8 (25.8) |
| ≥12 years | 645 (27.2) | 27 (12.3) | 1 (3.2) |
| Missing | 41 (1.7) | 13 (5.9) | 7 (22.6) |
| **Swedish background**** |  |  |  |
| Yes | 2 053 (86.6) | 187 (85.0) | 26 (83.9) |
| No | 313 (13.2) | 32 (14.5) | 5 (16.1) |
| Missing | 5 (0.2) | 1 (0.5) | 0 |
| **Cancer stage***** |  |  |  |
| N with index date ≥2000 | 1 660 | 185 | 31 |
| CS I | 937 (56.4) | 90 (48.6) | 13 (41.9) |
| CS II, III and Mk+**** | 426 (25.7) | 54 (29.2) | 11 (35.5) |
| CS IV | 267 (16.1) | 37 (20.0) | 6 (19.4) |
| Missing | 30 (1.8) | 4 (2.2) | 1 (3.2) |

Abbreviations: CS, Clinical stage; Mk+, marker positive.
Data are presented as numbers (%) unless otherwise specified.
*In years of schooling.
**Swedish background defined as born in Sweden with at least one parent born in Sweden.
***No overall stage distribution difference was noted between patients with neurodevelopmental disorders and those with no psychiatric diagnosis when tested with Fishers exact test (p=0.264)
****Due to few cases in each group CS II, CS III and Mk+ was merged in to one group in stage distribution analyses.

**Table S4** Clinical stage **in seminoma patients** with and without history of psychiatric diagnosis (online only)

| **Variable** | **No psychiatric diagnosis** | **Any psychiatric diagnosis** | **Neurodevelopmental disorders** |
| --- | --- | --- | --- |
| N | 3 250 | 325 | 39 |
| **Age** |  |  |  |
| Median (IQR) | 38 (32-45) | 38 (32-47) | 34 (27-39) |
| Range, min-max | 18-79 | 19-77 | 19-62 |
| *Categories* |  |  |  |
| 18-29 years | 582 (17.9) | 54 (16.6) | 14 (35.9) |
| 30-39 years | 1 278 (39.3) | 123 (37.8) | 16 (41.0) |
| 40-49 years | 831 (25.6) | 89 (27.4) | 6 (15.4) |
| ≥50 years | 559 (17.2) | 59 (18.2) | 3 (7.7) |
| **Index year** |  |  |  |
| 1992-2000 | 1 075 (33.1) | 68 (20.9) | 2 (5.1) |
| 2001-2008 | 1 173 (36.1) | 94 (28.9) | 8 (20.5) |
| 2009-2014 | 1 002 (30.8) | 163 (50.2) | 29 (74.4) |
| **Education*** |  |  |  |
| <9 years | 486 (15.0) | 94 (28.9) | 19 (48.7) |
| 10-12 years | 1 654 (50.9) | 158 (48.6) | 12 (30.8) |
| ≥12 years | 1 076 (33.1) | 62 (19.1) | 4 (10.3) |
| Missing | 34 (1.0) | 11 (3.4) | 4 (10.3) |
| **Swedish background**** |  |  |  |
| Yes | 2 866 (88.2) | 286 (88.0) | 35 (89.7) |
| No | 383 (11.8) | 39 (12.0) | 4 (10.3) |
| Missing | 1 (0.0) | (0.0) | (0.0) |
| **Cancer stage***** |  |  |  |
| N with index date ≥2000 | 2 308 | 262 | 37 |
| CS I | 1 892 (82.0) | 206 (78.6) | 26 (70.3) |
| CS II, III and Mk+**** | 265 (11.5) | 43 (16.4) | 8 (21.6) |
| **CS IV** | **28 (1.2)** | **7 (2.7)** | **2 (5.4)** |
| Missing | 123 (5.3) | 6 (2.3) | 1 (2.7) |

Abbreviations: CS, Clinical stage; Mk+ marker positive
Data are presented as numbers (%) unless otherwise specified.
*In years of schooling.
**Swedish background defined as born in Sweden with at least one parent born in Sweden.
***A significant difference in stage distribution overall was noted between patients with neurodevelopmental disorders and those with no psychiatric diagnosis when tested with Fishers exact test (p=0.036).
****Due to few cases in each group CS II, CS III and Mk+ was merged in to one group in stage distribution analyses.

**Table S5** RESTRICED TO INDEX YEAR ≥2001. Characteristics of cancer patients in subgroups of patients with non-seminoma and seminoma

| **Variable** | **Non-seminoma** | | | **Seminoma** | | | **Overall** | | |
| --- | --- | --- | --- | --- | --- | --- | --- | --- | --- |
|  | **Patients** | **Controls** | **p-value** | **Patients** | **Controls** | **p-value** | **Patients** | **Controls** | **p-value** |
| N | 1 736 | 17 360 |  | 2 432 | 24 320 |  | 4 168 | 41 680 |  |
| **Age** |  |  |  |  |  |  |  |  |  |
| Median (IQR) | 30 (24-36) | 30 (24-36) | 1.0 | 38 (32-45) | 38 (32-45) | 1.0 | 34 (28-42) | 34 (28-42) | 1.0 |
| Range, min-max | 18-78 | 18-78 |  | 18-79 | 18-79 |  | 18-79 | 18-79 |  |
| *Categories* |  |  |  |  |  |  |  |  |  |
| 18-29 years | 844 (48.6) | 8 440 (48.6) | 1.0 | 451 (18.5) | 4 510 (18.5) | 1.0 | 1 295 (31.1) | 12 950 (31.1) | 1.0 |
| 30-39 years | 584 (33.6) | 5 840 (33.6) |  | 959 (39.4) | 9 590 (39.4) |  | 1 543 (37.0) | 15 430 (37.0) |  |
| 40-49 years | 196 (11.3) | 1 960 (11.3) |  | 615 (25.3) | 6 150 (25.3) |  | 811 (19.5) | 8 110 (19.5) |  |
| ≥50 years | 112 (6.5) | 1 120 (6.5) |  | 407 (16.7) | 4 070 (16.7) |  | 519 (12.5) | 5 190 (12.5) |  |
| **Index year** |  |  |  |  |  |  |  |  |  |
| 2000-2008 | 934 (53.8) | 9 340 (53.8) | 1.0 | 1 267 (52.1) | 12 670 (52.1) | 1.0 | 2 201 (52.8) | 22 010 (52.8) | 1.0 |
| 2009-2014 | 802 (46.2) | 8 020 (46.2) |  | 1 165 (47.9) | 11 650 (47.9) |  | 1 967 (47.2) | 19 670 (47.2) |  |
| **Education*** |  |  |  |  |  |  |  |  |  |
| <9y | 307 (17.7) | 2 937 (16.9) | 0.11 | 312 (12.8) | 3 606 (14.8) | 0.005 | 619 (14.9) | 6 543 (15.7) | 0.002 |
| 10-12 years | 897 (51.7) | 9 091 (52.4) |  | 1 246 (51.2) | 12 713 (52.3) |  | 2 143 (51.4) | 21 804 (52.3) |  |
| ≥12 years | 494 (28.5) | 5 074 (29.2) |  | 842 (34.6) | 7 778 (32.0) |  | 1 336 (32.1) | 12 852 (30.8) |  |
| Missing | 38 (2.2) | 258 (1.5) |  | 32 (1.3) | 223 (0.9) |  | 70 (1.7) | 481 (1.2) |  |
| **Swedish background**** |  |  |  |  |  |  |  |  |  |
| Yes | 1 472 (84.8) | 14 545 (83.8) | 0.51 | 2 123 (87.3) | 20 319 (83.5) | <0.001 | 3 595 (86.3) | 34 864 (83.6) | <0.001 |
| No | 264 (15.2) | 2 813 (16.2) |  | 308 (12.7) | 3 998 (16.4) |  | 572 (13.7) | 6 811 (16.3) |  |
| Missing | 0 | 2 (0.0) |  | 1 (0.0) | 3 (0.0) |  | 1 (0.0) | 5 (0.0) |  |
| **Clinical stage** |  |  |  |  |  |  |  |  |  |
| CS I | 969 (55.8) |  |  | 2 012 (82.7) |  |  | 2 981 (71.5) |  |  |
| CS II | 369 (21.3) |  |  | 260 (10.7) |  |  | 629 (15.1) |  |  |
| CS III | 32 (1.8) |  |  | 34 (1.4) |  |  | 66 (1.6) |  |  |
| CS IV | 290 (16.7) |  |  | 34 (1.4) |  |  | 324 (7.8) |  |  |
| CS Mk+ | 45 (2.6) |  |  | 2 (0.1) |  |  | 47 (1.1) |  |  |
| Missing | 31 (1.8) |  |  | 90 (3.7) |  |  | 121 (2.9) |  |  |
| Missing <2000 | 0 |  |  | 0 |  |  | 0 |  |  |
| **Malformation and cryptorchidism** |  |  |  |  |  |  |  |  |  |
| Any malformation | 66 (3.8) | 556 (3.2) | 0.18 | 81 (3.2) | 583 (2.3) | 0.002 | 147 (3.5) | 1 132 (2.7) | 0.004 |
| Cryptorchidism | 44 (2.5) | 230 (1.3) | <0.001 | 61 (2.4) | 297 (1.2) | <0.001 | 103 (2.5) | 519 (1.2) | <0.001 |
| Any malformation (ICD) or cryptorchidism | 94 (5.4) | 693 (4.0) | 0.005 | 128 (5.0) | 792 (3.1) | <0.001 | 220 (5.3) | 1 470 (3.5) | <0.001 |

Abbreviations: IQR, interquartile range, CS, clinical stage; Mk+, marker positive
Data are presented as numbers (%) unless otherwise specified.
*In years of schooling.
**Swedish background defined as born in Sweden with at least one parent born in Sweden.

**Table S6** RESTRICED TO INDEX YEAR ≥2001. Odds ratios for the risk of germ cell testicular cancer by history of psychiatric disorders and malformation

| **Exposure** | **N subjects (% exposed)** | | **Model** | |
| --- | --- | --- | --- | --- |
|  | **Patients** | **Controls** | **Odds ratio (95% CI)** | **p-value** |
| **OVERALL** |  |  |  |  |
| Any psychiatric diagnosis | 437 (10.5) | 4 139 (9.9) | 1.06 (0.95-1.18) | 0.30 |
| Psychotic disorders | 38 (0.9) | 394 (0.9) | 0.96 (0.69-1.35) | 0.82 |
| Mood- and anxiety disorders | 195 (4.7) | 2 042 (4.9) | 0.95 (0.82-1.11) | 0.52 |
| Sleep disorders | 8 (0.2) | 78 (0.2) | 1.05 (0.51-2.17) | 0.90 |
| Eating and personality disorders | 37 (0.9) | 346 (0.8) | 1.08 (0.77-1.53) | 0.64 |
| Neurodevelopmental disorders | 66 (1.6) | 523 (1.3) | 1.18 (0.90-1.55) | 0.23 |
| Intellectual disabilities | 20 (0.5) | 153 (0.4) | 1.00 (0.61-1.65) | 0.98 |
| ADHD | 32 (0.8) | 250 (0.6) | 1.30 (0.89-1.88) | 0.17 |
| ASD | 21 (0.5) | 202 (0.5) | 0.96 (0.61-1.53) | 0.88 |
| ADHD or ASD | 48 (1.2) | 409 (1.0) | 1.16 (0.85-1.57) | 0.36 |
| Substance misuse | 168 (4.0) | 1 571 (3.8) | 1.10 (0.94-1.30) | 0.24 |
| **NON-SEMINOMA** |  |  |  |  |
| Any psychiatric diagnosis | 180 (10.4) | 1 723 (9.9) | 1.01 (0.85-1.19) | 0.93 |
| Psychotic disorders | 21 (1.2) | 141 (0.8) | 1.42 (0.89-2.27) | 0.14 |
| Mood- and anxiety disorders | 73 (4.2) | 826 (4.8) | 0.86 (0.67-1.10) | 0.22 |
| Sleep disorders | 3 (0.2) | 34 (0.2) | 0.88 (0.27-2.86) | 0.83 |
| Eating and personality disorders | 17 (1.0) | 145 (0.8) | 1.14 (0.69-1.90) | 0.61 |
| Neurodevelopmental disorders | 29 (1.7) | 285 (1.6) | 0.89 (0.60-1.33) | 0.58 |
| Intellectual disabilities | 10 (0.6) | 82 (0.5) | 0.92 (0.46-1.84) | 0.81 |
| ADHD | 16 (0.9) | 138 (0.8) | 1.10 (0.65-1.86) | 0.72 |
| ASD | 8 (0.5) | 115 (0.7) | 0.60 (0.29-1.24) | 0.16 |
| ADHD or ASD | 21 (1.2) | 225 (1.3) | 0.85 (0.54-1.35) | 0.50 |
| Substance misuse | 71 (4.1) | 625 (3.6) | 1.12 (0.87-1.45) | 0.36 |
| **SEMINOMA** |  |  |  |  |
| Any psychiatric diagnosis | 257 (10.6) | 2 416 (9.9) | 1.09 (0.95-1.25) | 0.22 |
| Psychotic disorders | 17 (0.7) | 253 (1.0) | 0.69 (0.42-1.13) | 0.14 |
| Mood- and anxiety disorders | 122 (5.0) | 1 216 (5.0) | 1.01 (0.84-1.23) | 0.88 |
| Sleep disorders | 5 (0.2) | 44 (0.2) | 1.20 (0.48-3.03) | 0.70 |
| Eating and personality disorders | 20 (0.8) | 201 (0.8) | 1.04 (0.65-1.65) | 0.87 |
| **Neurodevelopmental disorders** | **37 (1.5)** | **238 (1.0)** | **1.52 (1.06-2.19)** | **0.024** |
| Intellectual disabilities | 10 (0.4) | 71 (0.3) | 1.09 (0.53-2.23) | 0.81 |
| ADHD | 16 (0.7) | 112 (0.5) | 1.50 (0.88-2.55) | 0.13 |
| ASD | 13 (0.5) | 87 (0.4) | 1.51 (0.83-2.73) | 0.17 |
| **ADHD or ASD** | **27 (1.1)** | **184 (0.8)** | **1.52 (1.01-2.30)** | **0.045** |
| Substance misuse | 97 (4.0) | 946 (3.9) | 1.08 (0.87-1.34) | 0.47 |

Abbreviations: ADHD, Attention deficit activity disorders; ASD, Autism spectrum disorders;
***Model**: Conditioned on matching set (age and calendar year) and further adjusted for education, and congenital malformation or surgery for cryptorchidism

**Table S7** RESTRICED TO INDEX YEAR ≥2001. Risk of all-cause and testicular germ cell cancer-specific mortality overall and by subgroups in non-seminoma and seminoma with and without history of psychiatric diagnosis (hazard ratios with 95 confidence intervals (CI))

| **Group** | **N (%)** | | **N events** | | **Mortality rate (95% CI) per 1000 PY** | | **Model I**  **HR (95% CI)** | **Model II**  **HR (95% CI)** |
| --- | --- | --- | --- | --- | --- | --- | --- | --- |
|  | **Psychiatric diagnosis** | **No psychiatric diagnosis** | **Psychiatric diagnosis** | **No psychiatric diagnosis** | **Psychiatric diagnosis** | **No psychiatric diagnosis** |  |  |
| **All-cause morality** |  |  |  |  |  |  |  |  |
| Overall | 437 | 3 731 | 45 (10.3) | 153 (4.1) | 3.0 (2.1-3.9) | 1.2 (1.0-1.4) | **2.52 (1.79-3.55)** | **2.83 (1.98-4.05)** |
| Non-seminoma | 180 | 1 556 | 19 (10.6) | 68 (4.4) | 3.4 (1.9-5.0) | 1.5 (1.1-1.8) | **2.12 (1.24-3.63)** | **2.36 (1.36-4.12)** |
| Seminoma | 257 | 2 175 | 26 (10.1) | 85 (3.9) | 2.7 (1.7-3.8) | 1.1 (0.8-1.3) | **3.15 (1.99-5.00)** | **3.38 (2.08-5.52)** |
| **TGCC-specific mortality** |  |  |  |  |  |  |  |  |
| Overall | 437 | 3 731 | 17 (3.9) | 68 (1.8) | 5.2 (2.7-7.7) | 2.0 (1.5-2.4) | **2.15 (1.25-3.73)** | **2.12 (1.19-3.77)** |
| Non-seminoma | 180 | 1 556 | 11 (6.1) | 44 (2.8) | 8.0 (3.3-12.8) | 3.0 (2.1-3.9) | 1.97 (1.00-3.91) | 1.82 (0.88-3.75) |
| Seminoma | 257 | 2 175 | 6 (2.3) | 24 (1.1) | 3.1 (0.6-5.7) | 1.2 (0.7-1.7) | **3.20 (1.27-8.09)** | **3.27 (1.24-8.63)** |

Abbreviations: TGCC, testicular germ cell cancer; HR, hazard ratio **Model I:** Adjusted for age, index year, and education;

**Model II:** Model I and further adjusted for cancer stage (CS I, CS II+CSMk+, CS III+CS IV)

**Table S8** Cause of death among patients with or without history of psychiatric diagnosis

| **No psychiatric diagnosis** | | | |
| --- | --- | --- | --- |
|  | **Overall** | **Non-seminoma** | **Seminoma** |
| **Total, N event/N (%*)** | **393/5621 (7.0*)** | **151/2371 (6.4*)** | **242/3250 (7.4*)** |
| **Cause of death** |  |  |  |
| **All-cause of death** | **393 (100.0)** | **151 (100.0)** | **242 (100.0)** |
| Testicular cancer (C629 + 1869) | 133 (33.8) | 87 (57.6) | 46 (19.0) |
| Other cancers (C00-D48 except C629) | 92 (23.4) | 24 (15.9) | 68 (28.1) |
| Mental and behavioral disorders (F00-F99) | 3 (0.8) | <3** | <3** |
| External causes of morbidity and mortality (V01-Y98) | 19 (2.3) | 6 (4.0) | 13 (5.4) |
| Diseases of the circulatory or respiratory system (I00-J99) | 74 (18.8) | 19 (12.6) | 55 (22.7) |
| Other causes*** | 42 (10.7) | 7 (4.6) | 35 (14.5) |
| Missing | 30 (7.6) | 7 (4.6) | 23 (9.5) |
| **Psychiatric diagnosis** | | | |
|  | **Overall** | **Non-seminoma** | **Seminoma** |
| **Total, N events/N (%*)** | **80/545 (14.7*)** | **29/220 (13.2*)** | **51/325 (15.7*)** |
| **Cause of death** |  |  |  |
| **All-cause of death** | **80 (100.0)** | **29 (100.0)** | **51 (100.0)** |
| Testicular cancer (C629 + 1869) | 24 (30.0) | 14 (48.2) | 10 (19.6) |
| Other cancers (C00-D48 except C629) | 12 (15.0) | 3 (10.3) | 9 (17.6) |
| Mental and behavioral disorders (F00-F99) | 5 (6.3) | <5** | **<5**** |
| External causes of morbidity and mortality (V01-Y98) | 13 (16.3) | 5 (17.2) | 8 (15.7) |
| Diseases of the circulatory or respiratory system (I00-J99) | 10 (12.5) | <3** | 8 (15.7) |
| Other causes*** | 8 (10.0) | 0 (0.0) | 8 (15.7) |
| Missing | 8 (10.0) | 4 (13.8) | 4 (7.8) |

Data are presented as numbers (%) unless otherwise specified.
*% deceased of included patients
**Less than 3 patients not presented due to potentially reconductable to single individuals.
***Other causes: Certain infectious and parasitic diseases (A00-B99), Endocrine, nutritional and metabolic diseases (E00-E90), Diseases of the nervous system (G00-G99), Diseases of the digestive system (K00-K93), Diseases of the musculoskeletal system and connective tissue (M00-M99), Diseases of the genitourinary system (N00-N99), Congenital malformations, deformations and chromosomal abnormalities (Q00-Q99), Symptoms, signs and abnormal clinical and laboratory findings, not elsewhere classified (R00-R99).
